# A new decoding algorithm for hidden Markov models improves the prediction of the topology of all-beta membrane proteins

**DOI:** 10.1186/1471-2105-6-S4-S12

**Published:** 2005-12-01

**Authors:** Piero Fariselli, Pier Luigi Martelli, Rita Casadio

**Affiliations:** 1 Department of Biology, University of Bologna via Irnerio 42, 40126 Bologna, Italy

## Abstract

**Background:**

Structure prediction of membrane proteins is still a challenging computational problem. Hidden Markov models (HMM) have been successfully applied to the problem of predicting membrane protein topology. In a predictive task, the HMM is endowed with a *decoding algorithm *in order to assign the most probable state path, and in turn the labels, to an unknown sequence. The Viterbi and the posterior decoding algorithms are the most common. The former is very efficient when one path dominates, while the latter, even though does not guarantee to preserve the HMM grammar, is more effective when several concurring paths have similar probabilities. A third good alternative is 1-best, which was shown to perform equal or better than Viterbi.

**Results:**

In this paper we introduce the posterior-Viterbi (PV) a new decoding which combines the posterior and Viterbi algorithms. PV is a two step process: first the posterior probability of each state is computed and then the best posterior allowed path through the model is evaluated by a Viterbi algorithm.

**Conclusion:**

We show that PV decoding performs better than other algorithms when tested on the problem of the prediction of the topology of beta-barrel membrane proteins.

## Background

All-beta membrane proteins constitute a well structurally conserved class of proteins, that span the outer membrane of Gram-negative bacteria with a barrel-like structure. In all cases known so far with atomic resolution, the barrel consists of an even number of anti-parallel beta strands, whose number ranges from 8 to 22 strands, depending on the protein and/or its functional role [1,2]. In eukaryotes, it is known that similar architectures are present in the outer membrane of chloroplasts and mitochondria, although so far none of the so-called "porins", mainly acting as Voltage Dependent Anion Channels (VDAC), have been solved with atomic resolution ([3] and references therein). It is therefore urgent to devise methods for the prediction of the topology of this class of membrane proteins. Indeed the correct prediction of the protein topology, given the conservation of the barrel architecture may greatly help in threading procedures, especially when sequence homology is low. Furthermore reliable methods, endowed with a low rate of false positives, can also help in genome annotation on the basis of protein structure prediction [3,4]. The problem of predicting beta barrel membrane proteins has been recently addressed with machine learning approaches, and among them Hidden Markov Models (HMMs) have been shown to outperform previously existing methods [5]. HMMs were developed for alignments [6,7], pattern detection [8,9] and also for predictions, as in the case of the topology of all-alpha and all-beta membrane proteins [10-17]. When HMMs are implemented for predicting a given feature, a *decoding *algorithm is needed. With decoding we refer to the assignment of a path through the HMM states (which is the best under *a suitable measure*) given an observed sequence *O*. In this way, we can also assign a label to each sequence element [18,19]. More generally, as stated in [20], the decoding is the prediction of the labelsof an unknownpath. The most famous decoding procedure is the Viterbi algorithm, which finds the most probable allowed path through the HMM model. Viterbi decoding is particularly effective when there is a *single best path *among others much less probable. When several paths have similar probabilities, the posterior decoding or the 1-best algorithms are more convenient [20]. The posterior decoding assigns the state path on the basis of the posterior probability, although the selected path might be not allowed.

In this paper we address the problem of preserving the automaton grammar and concomitantly exploiting the posterior probabilities, without the need of the post-processing algorithm [12,21]. Prompted by this, we design a new decoding algorithm, the posterior-Viterbidecoding (PV), which preserves the automaton grammars and at thesame time exploits the posterior probabilities. A related idea, that is specific for pairwise alignments was previously introduced to improve the sequence alignment accuracy [22]. We show that PV performs better than the other algorithms when we test it on the problem of predicting the topology of beta-barrel membrane proteins.

## Results and Discussion

### Testing the decoding algorithms on all-beta membrane proteins

In order to test our decoding algorithm on real biological data, we used a previously developed HMM, devised for the prediction of the topology of beta-barrel membrane proteins [12]. The hidden Markov model is a sequence-profile-based HMM and takes advantage of emitting vectors instead of symbols, as described in [12].

Since the previously designed and trained HMM [12] emits profile vectors, sequence profiles have been computed from the alignments as derived with PSI-BLAST [23] on the non-redundant database of protein sequences .

The results obtained using the four different decoding algorithms are shown in Table 1, where the performance is tested with a leave-one-out cross validation procedure for the first 15 proteins and as blind-test for the latter 5 (see Methods). It is evident that for the problem at hand the Viterbi decoding and the 1-best are unreliable, since only one of the proteins is correctly assigned. In this case the posterior decoding is more efficient and can correctly assign 60% and 40% of the proteins, in cross-validation and on the blind set, respectively. Here the posterior decoding is used without MaxSubSeq, introduced before to recast the grammar [12].

From Table 1 it evident that the new PV decoding is the best performing decoding achieving 80% and 60% accuracy in cross-validation and on the blind set, respectively. This is done ensuring that predictions are consistent with the designed automaton grammar.

### Comparison with other available HMMs

In Table 2 we show the results of the comparison between our HMM-decoding with those obtained from the available web servers, based on similar approaches [16,17,21]. The **pred-tmbb **server [16] allows the user to test three different algorithms (namely Viterbi, 1-best and posterior). Differently from us they find that their HMM does not show significant differences among the three decoding algorithms. This dissimilar behaviour may be due to several concurring facts: *i*) the different HMM models, *ii*) **pred-tmbb **runs on a single-sequence input, *iii*) **pred-tmbb **is trained using the *Conditional Maximum Likelihood *[24]. The second server **PROFtmb **[17] is based on a method that exploits multiple sequence information and posterior probabilities. Their decoding is related to the posterior-Viterbi; however, in their algorithm the authors first obtained the posterior sum contracted into two possible labeling (inner/outer loops and transmembrane as we did in [12]), then they made use of the explicit value of the HMM transition probabilities (*a*_*i*,*j*_). In this way they count the transition probabilities twice (implicitly in the posterior-probability and directly into their algorithm) and the **PROFtmb **performance is not very different from ours.

Finally, the third server **HMMB2TMR **[21] achieves a performance quite similar to that obtained with PV decoding. To do that **HMMB2TMR **takes advantage of the MaxSubSeq algorithm on top of the posterior sum decoding. However, although MaxSubSeq is a very general two-class segment optimization algorithm, it is a post processing procedure that has to be applied after a HMM decoding. On the contrary, PV is a general decoding algorithm and it is more useful when the underlying predictor is a HMM, where more than two labels and different constraints can be introduced into the automaton grammars.

## Conclusion

The new PV decoding algorithm is more convenient in that it overcomes the difficulties of introducing a problem-dependent optimization algorithm when the automaton grammar is to be re-cast. When one-state-path dominates we may expect that PV does not perform better than the other decoding algorithms, and in these cases the 1-best is preferred [20]. Nevertheless, we show that when several concurring paths are present, as in the case of our beta-barrel HMM, PV performs better than the others. Although PV takes more time than other algorithms (the posterior + the Viterbi time), the PV asymptotic computational time-complexity still remains *O*(*N*^2^·*L*) (where *L *and *N *are the protein length and the number of states, respectively) as for the other decodings. As far as the memory requirement is concerned, PV needs the same space-complexity of the Viterbi and posterior (*O*(*N*·*L*)), while 1-*best *in the average case requires less memory, and can also be reduced [20]. When computational speed is an issue, Viterbi algorithm is the fastest and the time complexity order is *time*(*viterbi*) ≤ *time*(l - *best*) ≤ *time*(*PV*). Finally, PV satisfies any HMM grammar structures, including automata containing silent states, and it is applicable to all the possible HMM models with an arbitrary number of labels and without having to work out a problem-dependent optimization algorithm.

## Methods

### The hidden Markov model definitions

For sake of clarity and compactness, in what follows we make use of explicit *BEGIN *(*B*) and *END *states and we do not treat the case of the *silent (null) *states. However, their inclusion in the algorithms is only a technical matter and can be done following the prescriptions indicated in [18,19].

An observed sequence of length *L *is indicated as *O *(= *O*_1_...*O*_*L*_) both for a single-symbol-sequence (as in the standard HMMs) or for a vector-sequence as described before [12]. *λ*(*s*) indicates the label associated to the state *s*, while Λ (= Λ_*i*_,... Λ_*L*_) is the list of the labels associated to each sequence position *i *obtained after the application of a decoding algorithm. Depending on the problem at hand, the labels may identify transmembrane regions, loops, secondary structures of proteins, coding/non coding regions, intergenic regions, etc. A HMM consisting of *N *states (indicated below with *s *and *k*) is therefore defined by three probability distributions:

Starting probabilities

*a*_*B*,*k *_= *P*(*k*|*B*)     (1)

Transition probabilities

*a*_*k*,*s *_= *P*(*s*|*k*)     (2)

Emission probabilities

*e*_*k*_(*O*_*i*_) = *P*(*O*_*i*_|*k*)     (3)

The forward probability is

*f*_*k*_(*i*) = *P*(*O*_1_,*O*_2_...*O*_*i*_,*π*_*i *_= *k*)     (4)

which is the probability of having emitted the first partial sequence up to position *i *ending at state *k*. The backward probability is:

*b*_*k*_(*i*) = *P*(*O*_*i*+1_,... *O*_*L *- 1_, *O*_*L*_|*π*_*i *_= *k*)     (5)

which is the probability of having emitted the sequence starting from the last element back to the (*i*+l)th element, given that we end at position *i *in state *k*. The probability of emitting the whole sequence can be computed using either the forward or backward probabilities according to:

*P*(*O*|*M*) = *f*_*END*_(*L *+ 1) = *b*_*B*_(0)     (6)

Forward and backward probabilities are also necessary for updating the HMM parameters, using the Baum-Welch algorithm [18,19]. Alternatively a gradient-based training algorithm can be applied [18,20].

### Viterbi decoding

Viterbi decoding finds the path (*π*) through the model which has the maximal probability [18,19]. This means that we look for the path which is

*π*^*v *^= *argmax*_{*π*}_*P*(*π*|*O*, *M*)     (7)

where *O*(= *O*_1_,... *O*_*L*_) is the observed sequence of length *L *and *M *is the trained HMM model. Since the *P*(*O*|*M*) is independent of a particular path *π*, Equation 7 is equivalent to

*π*^*v *^= *argmax*_{*π*}_*P*(*π*, *O*|*M*)     (8)

*P*(*π*, *O*|*M*) can be easily computed as



where by construction *π*(0) is always the *BEGIN *state (*B*).

Defining *v*_*k*_(*i*) as the probability of the most likely path ending in state *k *at position *i*, and *p*_*i*_(*k*) as the trace-back pointer, *π*^*v *^can be obtained running the following dynamic programming algorithm called Viterbi decoding:

• Initialization

*v*_*B*_(0) = 1 *v*_*k*_(0) = 0 *for k *≠ *B*

• Recursion



• Termination



• Traceback



• Label assignment



where *λ*(*s*) is the label associated to the *s *state.

### 1-best decoding

The 1-best labeling algorithm described here is Krogh's previously described variant of the N-best decoding [20]. Since there is no exact algorithm for finding the most probable labeling, 1-best is an approximate algorithm which usually achieves good results in solving this task [20]. Differently from Viterbi, the 1-best algorithm ends when the most probable labeling is computed, so that no trace-back is needed.

For sake of clarity, here we present a redundant description, in which we define *H*_*i *_as the set of all labeling hypotheses surviving as 1-best for each state *s *up to sequence position *i*. In the worst case the number of distinct labeling-hypotheses is equal to the number of states,  is the current partial labeling hypothesis associated to the *s *state from the beginning to the *i*-th sequence position. In general several states may share the same labeling hypothesis. Finally, we use ⊕ as the *string concatenation operator*, so that 'AAAA'⊕'B' = 'AAAAB' (the empty string is " and the empty set is ∅). Thus 1-best algorithm can be described as

• Initialization

*v*_*B*_(*"*) = 1 *v*_*k*_(*"*) = 0 *for k *≠ *B*

*v*_*k*_(*λ*(*k*)) = *a*_*B*,*k*_·*e*_*k*_(*O*_1_)

*H*_1 _= {*λ*(*k*) : *a*_*B*,*k *_≠ 0} *H*_*i *_= ∅ *for i *≠ 1

• Recursion



• Termination



With 1-best decoding, we do not need to keep a backtrace matrix since Λ is computed during the forward steps.

### Posterior decoding

The *posterior *decoding finds the path which maximizes the product of the *posterior *probability of the states [18,19]. Using the usual notation for forward (*f*_*k*_(*i*)) and backward (*b*_*k*_(*i*)) we have

*P*(*π*_*i *_= *k*|*O*,*M*) = *f*_*k*_(*i*)*b*_*k*_(*i*)/*P*(*O*|*M*)     (10)

The path *π*^*p *^which maximizes the posterior probability is then computed as



for *i = *1... *L*. The corresponding label assignment is



If we have more than one state sharing the same label, labeling can be improved by summing over the states that share the same label *(posterior sum*). In this way we can have a path through the model which maximizes the posterior probability of being in a state with *label λ *when emitting the observed sequence element, or more formally:



Λ_*i *_= *argmax*_{*λ*}_*P*(*label*(*O*_*i*_) = *λ *|*O*, *S*)     (14)

where *i *ranges from 1 to *L*.

The posterior-decoding drawback is that the state path sequences *π*^*p *^or Λ may be not feasible paths.

However, this decoding can perform better than Viterbi, when more than one highly probable path exists [18,19]. In this case a post-processing algorithm that recasts the original topological constraints is recommended [21].

In the sequel, if not differently indicated, with the term *posterior *we mean the posterior sum.

### Posterior-Viterbi decoding

Posterior-Viterbi decoding is based on the combination of the Viterbi and posterior algorithms. After having computed the *posterior *probabilities we use a Viterbi algorithm to find the best *allowed posterior *path through the model. A related idea, specific for pairwise alignments was previously introduced to improve the sequence alignment accuracy [22].

In the PV algorithm, the basic idea is to compute the path *π*^*PV*^



where *A*_*p *_is the set of the allowed paths through the model, and *P*(*π*_*i*_|*O*,*M*) is the *posterior *probability of the state assigned by the path *π *at position *i *(as computed in Eq. 10).

Defining a function δ*(*s*, *t*) equal to 1 if *s *→ *t *is an allowed transition of the model *M*, 0 otherwise; *v*_*k*_(*i*) as the probability of the most probable *allowed-posterior *path ending at state *k *having observed the partial *O*_1_,... *O*_*i *_and *p*_*i *_as the trace-back pointer, we can compute the best path *π*^*PV *^using the Viterbi algorithm:

• Initialization

*v*_*B*_(0) = 1 *v*_*k*_(0) = 0 *for k *≠ *B*

• Recursion



• Termination



• Traceback



• Label assignment



An alternative approach, that directly maximizes the most probable labelling, is to substitute the posterior probability of a given state *P*(*π*_*i *_= *k*|*O*, *M*), with the posterior sum *P*(*label*(*O*_*i*_) = *λ*|*O*, *M*) (equation 14). In this case all the states that share the same label have the same probability for each sequence position. However, since the performances of this second version are slightly worse we do not show them.

### Datasets

The problem of the prediction of the all-beta transmembrane regions is used to test the algorithm on a real data application. In this case we use a set that includes 20 constitutive beta-barrel membrane proteins whose sequences are less than 25% homologous and whose 3D structure have been resolved. The number of beta-strands forming the transmembrane barrel ranges from 2 to 22. Among the 20 proteins, 15 were used to train a circular HMM (described in [12]), and here are tested in cross-validation (1a0sP, 1bxwA, 1e54, 1ek9A, 1fcpA, 1fep, 1i78A, 1k24, 1kmoA, 1prn, 1qd5A, 1qj8A, 2mprA, 2omf, 2por). Since there is no detectable sequence identity among the selected 15 proteins, we adopted a leave-one-out approach for training the HMM and testing it. All the reported results are obtained during the testing phase, and the complete set of results is available at . The other 5 new proteins (1mm4, 1nqf, 1p4t, 1uyn, 1t16) are used as a blind new test. Since our goal is to predict the beta-strands that span the membrane we score the methods using the annotations derived from the PDB files. An alternative approach not addressed here, is to predict the portion of the transmembrane beta-strands in contact with the lipid bilayer. This prediction is however out of the scope of our approach, since in real porins the localization of the beta-strands in contact with the membrane, has been so far estimated by means of different computational methods and assumptions [25].

### Measures of accuracy

We used three indices to score the accuracy of the algorithms. The first one is *Q*_2 _which computes the number of correctly assigned labels divided by the total number of observed symbols. Then we use the *SOV *index [26] to evaluate the segment overlaps. Finally, we also adopt a very stringent measure called *Q*_*ok *_: a prediction is considered correct *only if the number of transmembrane segments coincides with the observed one and the corresponding segments have a minimal overlap of m residues *[21]. The value *m *is segment-dependent and for each segment pairs, is computed as

*m *= *min*{|*seg*_*pr*_*|/*2, |*seg*_*ob*_|/2}     (16)

where |*seg*_*pr*_| and |*seg*_*ob*_| are the predicted and observed segment lengths, respectively.

## List of abbreviations

• HMM: hidden Markov model.

• PV: Posterior-Viterbi.

• *B: *Begin state.

## Authors' contributions

PF developed the Posterior-Viterbi algorithm. PLM designed and trained the Hidden Markov Models. RC contributed to the problem. PF, PLM and RC authored the manuscript.

## Figures and Tables

**Table 1:** *Q*_*ok *_accuracy obtained with the four different decoding algorithms

Proteins	Viterbi	1-best	posterior	posterior-Viterbi
*cross-validation*				
1a0spTOT	-	-	-	OK
1bxwaTOT	-	OK	OK	OK
1e54	-	-	OK	OK
lek9aTOT	-	-	OK	OK
1fcpaTOT	-	-	-	-
1fepTOT	-	-	-	OK
1i78a	-	-	OK	OK
1k24	-	-	-	OK
1kmoaTOT	-	-	OK	OK
1prn	-	-	-	-
1qd5a	-	-	OK	OK
1qj8a	-	-	OK	OK
2mpra	-	-	OK	OK
2omf	-	-	OK	OK
2por	-	-	-	-
<*Q*_*ok *_>	0.0	0.07	0.60	0.80
				
*blind-test*				
1mm4	-	-	OK	-
1nqf	-	-	-	OK
1p4t	OK	OK	OK	OK
1uyn	-	-	-	OK
1t16	-	-	-	-
<*Q*_*ok *_>	0.20	0.20	0.40	0.60

**Table 2:** PV accuracy compared with other algorithms and HMM models

Method	*Q*_2_	SOV	SOV(BetaTM)	SOV(Loop)	*Q*_*ok*_
*cross-validation*					
Posterior-Viterbi^l^	0.82	0.87	0.92	0.81	0.80
Viterbi^1^	0.63	0.33	0.27	0.35	0.0
1-best^1^	0.65	0.41	0.37	0.41	0.07
HMMB2HTMR^2^	0.83	0.87	0.88	0.84	0.73
PROFTmb^3^	0.83	0.87	0.88	0.84	0.73
pred-tmbb^4 ^(Viterbi)	0.78	0.83	0.81	0.82	0.60
pred-tmbb^4 ^(1-best)	0.78	0.83	0.81	0.82	0.60
pred-tmbb^4 ^(posterior)	0.78	0.82	0.80	0.82	0.60
					
*blind-test*					
Posterior-Viterbi^1^	0.80	0.81	0.84	0.74	0.60
Viterbi^1^	0.62	0.38	0.35	0.40	0.20
1-best^1^	0.63	0.38	0.36	0.40	0.20
HMMB2HTMR^2^	0.80	0.81	0.84	0.74	0.60
PROFTmb^3^	0.72	0.65	0.72	0.58	0.40
pred-tmbb^4 ^(Viterbi)	0.71	0.73	0.79	0.71	0.20
pred-tmbb^4 ^(1-best)	0.71	0.73	0.79	0.71	0.20
pred-tmbb^4 ^(posterior)	0.72	0.75	0.81	0.71	0.20

1 Model taken from Martelli et al., 2002 [12]

2 Fariselli et al. 2003 [21]

3 Bigelow et al., 2004 [17]

4 Bagos et al., 2004 [16]
